# Hybrid Push-Pull Endoscopic and Laparoscopic Full Thickness Resection for the Minimally Invasive Management of Gastrointestinal Stromal Tumors: A Pilot Clinical Study

**DOI:** 10.1155/2015/618756

**Published:** 2015-04-06

**Authors:** Field F. Willingham, Paul Reynolds, Melinda Lewis, Andrew Ross, Shishir K. Maithel, Flavio G. Rocha

**Affiliations:** ^1^Division of Digestive Diseases, Department of Medicine, Emory University School of Medicine, Atlanta, GA 30322, USA; ^2^Division of Cytology, Department of Pathology, Emory University School of Medicine, Atlanta, GA 30322, USA; ^3^Section of Gastroenterology, Department of Medicine, Digestive Disease Institute, Virginia Mason Medical Center, Seattle, WA 98101, USA; ^4^Division of Surgical Oncology, Department of Surgery, Emory University School of Medicine, Atlanta, GA 30322, USA; ^5^Section of General, Thoracic and Vascular Surgery, Department of Surgery, Digestive Disease Institute, Virginia Mason Medical Center, Seattle, WA 98101, USA

## Abstract

*Background*. Gastric gastrointestinal stromal tumors (GISTs) that are predominantly endophytic or in anatomically complex locations pose a challenge for laparoscopic wedge resection; however, endoscopic resection can be associated with a positive deep margin given the fourth-layer origin of the tumors. *Methods*. Patients at two tertiary care academic medical centers with gastric GISTs in difficult anatomic locations or with a predominant endophytic component were considered for enrollment. Preoperative esophagogastroduodenoscopy (EGD), endoscopic ultrasound (EUS) with or without fine needle aspiration (FNA), and cross-sectional imaging were performed. Eligible patients were offered and consented for hybrid and standard management. *Results*. Over ten months, four patients in two institutions with anatomically complex or endophytic GISTs underwent successful, uncomplicated push-pull hybrid procedures. GIST was confirmed in all resection specimens. *Conclusion*. In a highly selected population, the hybrid push-pull approach was safe and effective in the removal of complex gastric GISTs. Endoscopic resection alone was associated with a positive deep margin, which the push-pull technique manages with a laparoscopic, full thickness, R0 resection. This novel, minimally invasive, hybrid laparoscopic and endoscopic push-pull technique is a safe and feasible alternative in the management of select GISTs that are not amenable to standard laparoscopic resection.

## 1. Introduction

Gastrointestinal stromal tumors (GISTs) are the most common mesenchymal tumors involving the gastrointestinal tract, with an estimated annual incidence between 4000 and 6000 new cases per year in the United States [1–3]. While targeted inhibitor therapy with agents such as imatinib is selectively employed in adjuvant and neoadjuvant settings, surgical resection remains the standard of care for patients with localized disease [4, 5]. A wedge resection is often the approach of choice for tumors of gastric origin, especially for exophytic tumors and those involving the body of the stomach (as nodal harvest is typically not required). More recently, laparoscopic approaches to gastric GIST resection have been employed, especially in exophytic tumors along the greater curvature of the stomach. These approaches have had equivalent outcomes to open procedures and suggest faster recovery times [6]. However, endophytic tumors may be difficult to locate laparoscopically and may require additional gastrotomies or substantial resections, incorporating a large proportion of the surrounding gastric wall in order to obtain a negative margin. Tumors involving the cardia and gastroesophageal (GE) junction may require a total gastrectomy, which can be a highly morbid surgery with life-long quality-of-life implications [7]. Tumors involving the pyloric channel may require a formal distal gastrectomy with a Billroth type reconstruction which, while better tolerated than a total gastrectomy, can lead to dumping syndrome, bile reflux gastritis, and an increased risk of gastric stump carcinoma [8–11].

Endoscopic resection has also been employed for submucosal mass lesions involving the stomach [12, 13]. Endoscopy is safe and effective with a very low rate of complications and little to no postoperative morbidity. Endoscopic resection is, however, more applicable to small tumors that are superficial to the muscularis propria layer. GISTs often arise from the muscularis propria layer and thus are more difficult to resect endoscopically as they do not readily constrain above an endoscopic snare or lift with submucosal injection. While endoscopic resection of tumors from the fourth layer (muscularis propria) has been described [14], there is concern that endoscopic resection may divide across, and not below, the deep margin of the tumor. Thus, it is likely that, for fourth layer mass lesions, tumor may be left behind at the base of the resection site.

We have previously described a combined approach for the hybrid endoscopic and laparoscopic management of mass lesions involving the foregut [7]. This approach has been highly effective in a select group of patients. For fourth-layer GISTs, we herein examine a push-pull modification of the hybrid approach which allows endoscopic resection of the tumor with laparoscopic assistance (push) followed by full thickness laparoscopic resection of the tumor base with endoscopic assistance (pull). This push-pull technique may provide a safe, minimally invasive alternative for resection of fourth-layer tumors, especially those that are predominantly endophytic or which arise in challenging anatomical locations, while still providing an oncologically sound procedure. This study examines the outcomes for patients in two academic medical centers that have undergone a hybrid push-pull endoscopic and laparoscopic resection for gastric GIST tumors.

## 2. Materials and Methods

### 2.1. Study Design

This pilot clinical study enrolled a nonrandomized cohort of patients from two tertiary care academic medical centers. The study was approved by the Institutional Review Boards (IRB) of both institutions. The standard and hybrid approaches were reviewed and discussed in detail with patients. All patients provided written informed consent for the hybrid endoscopic and laparoscopic procedure, as well as the corresponding standard surgical approach in case the hybrid procedure could not be completed as planned. The primary aim of the study was to examine the patient selection, procedural characteristics, pathologic details, and postoperative course for patients undergoing hybrid endoscopic and laparoscopic surgery for anatomically complex gastric GISTs. All data regarding preoperative pathologic diagnoses, presurgical imaging and endoscopic evaluation, operative details, final pathologic diagnoses, and postoperative course were abstracted from electronic patient records. Data was collected for patients offered hybrid management from September 2012 to May 2013. All hybrid procedures were performed by one attending surgeon (Flavio G. Rocha and Shishir K. Maithel) and one attending gastroenterologist (Andrew Ross and Field F. Willingham).

### 2.2. Patient Selection

Patients were considered for push-pull hybrid management when they had a submucosal tumor that was confirmed to be a GIST, that was not thought to be amenable to endoscopic resection, and that had anatomic features precluding a standard laparoscopic gastric wedge resection. Preoperative esophagogastroduodenoscopy (EGD), endoscopic ultrasound (EUS) with biopsy and/or FNA, and cross-sectional imaging were reviewed. Each patient was discussed at GI tumor board on a case-by-case basis. Options such as endoscopic submucosal dissection (ESD) and standard surgical management were discussed in addition to the hybrid technique. Those with potentially amenable lesions were considered for the push-pull hybrid approach. All management options, risks, benefits, and alternatives were discussed with the patients in detail. Patients had to be candidates for surgery based on review of their performance status and comorbid conditions. After meeting these strict inclusion criteria, no patients were excluded.

### 2.3. Description of Procedure

All procedures were performed in the operating room under general anesthesia with patients in a supine position. The abdomen was prepped and draped in standard sterile fashion. The endoscopist was positioned at the patient's head. A 10 mm periumbilical incision was made; the peritoneal cavity was entered, and a Hasson trocar was placed for visualization of the abdomen. Insufflation using carbon dioxide was performed to establish pneumoperitoneum and a four-quadrant laparoscopic examination was performed. Two or three additional 5 mm or 10 mm transabdominal ports were placed under direct visualization as needed. Port placement varied by tumor location. The gastrocolic ligament was divided and the lesser sac entered to access tumors in the posterior gastric wall. The stomach was exposed and was mobilized laparoscopically by the surgeons as necessary per case.

The esophagus was intubated with a standard single channel endoscope (Olympus America, Center Valley, PA) and the gastric tumor was visualized. The surgeon, using assistance from the endoscopic view of the tumor, invaginated the tumor into the gastric lumen using laparoscopic atraumatic bowel graspers to push on the exterior aspect of the mass via the serosal surface of the gastric wall. The gastroenterologist then constrained the tumor within either a 5.5 cm (Cook Medical, Bloomington, IN) or a 33 mm (Captivator, Boston Scientific, Natick, Massachusetts) needle tip endoscopic electrocautery snare. The gastric mass was then resected using electrocautery. The resected mass lesions were retrieved perorally using a Roth Net (US Endoscopy, Mentor, OH). The surgical team was prepared to perform laparoscopic closure if a full thickness defect occurred and also was prepared for the placement of intragastric trocars if needed for manipulation of the tumor. The endoscopic team was prepared for management of bleeding from the site with cautery or endoscopic clips as needed.

Following the endoscopic retrieval of the specimen, the base of the lesion was approached again using laparoscopic and endoscopic imaging to grasp the gastric wall overlying the resection site. The endoscopist pushed the resection site into the jaws of the bowel graspers with an endoscopic catheter. The laparoscopic stapler was then introduced via the Hasson trocar with the guidance of a 5 mm camera. Using laparoscopic and endoscopic visualization, the gastric wall overlying the endoscopic resection site was manipulated into the jaws of the stapler. Paying careful attention to the resection margin and to maintaining a patent gastric lumen, the gastric wall was divided in a full thickness manner to remove the resection site with a small margin of surrounding gastric wall. The resection site was then placed into a specimen retrieval bag and removed through the periumbilical Hasson port. The stomach was then carefully examined again endoscopically to ensure there was no bleeding and that the lumen had not been compromised. The operative field was then carefully examined laparoscopically to ensure that there was no air leak from the resection site. All of the sponge and instrument counts were checked per standard operative protocol. The patient was extubated and transferred to the recovery area for postoperative monitoring.

## 3. Results

**Figure 1:**
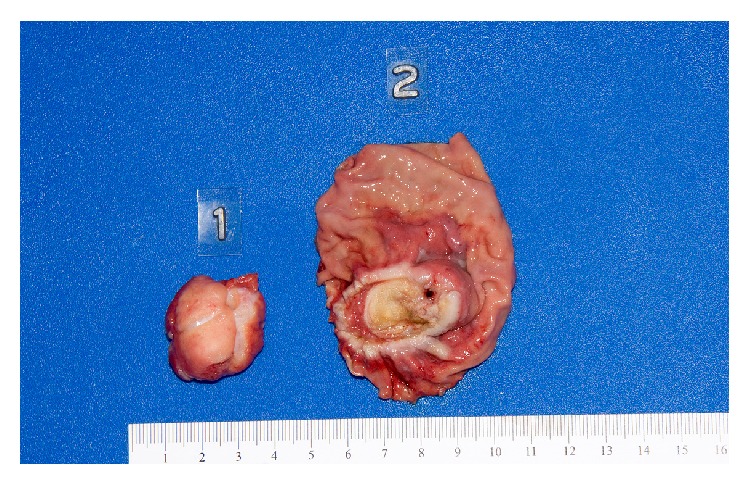
Gross pathologic evaluation of resected GIST using push-pull technique. Specimen 1 is the tumor resected endoscopically while Specimen 2 is the corresponding base of the tumor removed by subsequent laparoscopic wedge resection.

Between September 2012 and May 2013, four patients were selected for hybrid push-pull resection. The four patients ranged in age from 56 to 75 years of age (see Table 1); two were male. The first patient presented with melena and anemia. EUS demonstrated an endophytic submucosal mass arising from the muscularis propria layer (4.7 × 2.6 cm) located close to the GE junction. EUS biopsy revealed a diagnosis of GIST. After multidisciplinary discussion, the patient was treated with imatinib neoadjuvant therapy for six months in an effort to reduce the size of the tumor and was then taken to the operating room for the hybrid resection. The lesion was resected endoscopically with laparoscopic assistance. Full thickness gastric wedge resection was then performed laparoscopically without impingement on the GE junction. The resected tumor measured 3.0 and 2.8 cm in longest dimensions (see Figure 1). The mitotic rate was 1/50 high-power fields (hpf). Given the original size and unknown mitotic rate prior to imatinib therapy, the decision was made to continue adjuvant therapy for an additional 2.5 years.

**Figure 2:**
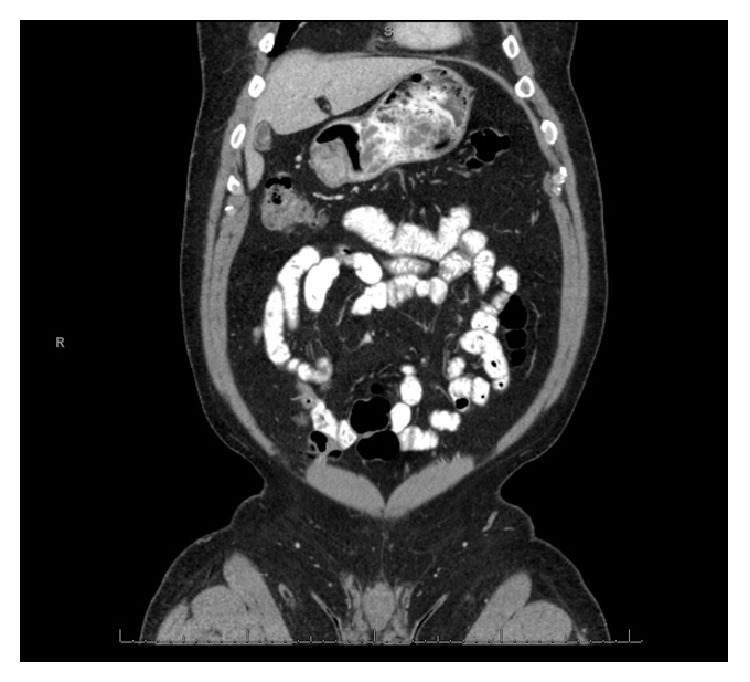
Image from a computed tomography scan demonstrating an endophytic tumor arising in a challenging location in the gastric antrum.

The second patient presented with dysphagia and underwent a computed tomography (CT) scan (Figure 2) and subsequent EGD which demonstrated an endophytic 4 cm submucosal mass in the antrum of the stomach. Superficial biopsies were nondiagnostic. An EUS was performed. The case was reviewed and the patient was brought for a hybrid push-pull resection. Due to the highly endophytic nature of the mass, it was resected endoscopically in two sections. The latter resection required a full thickness excision resulting in a gastrotomy. This was closed laparoscopically with a stapler. Pathologic examination of the specimen demonstrated a tumor approximately 4.2 cm in length with a mitotic rate of 1 mitosis/50 hpf and final negative margins.

**Figure 3:**
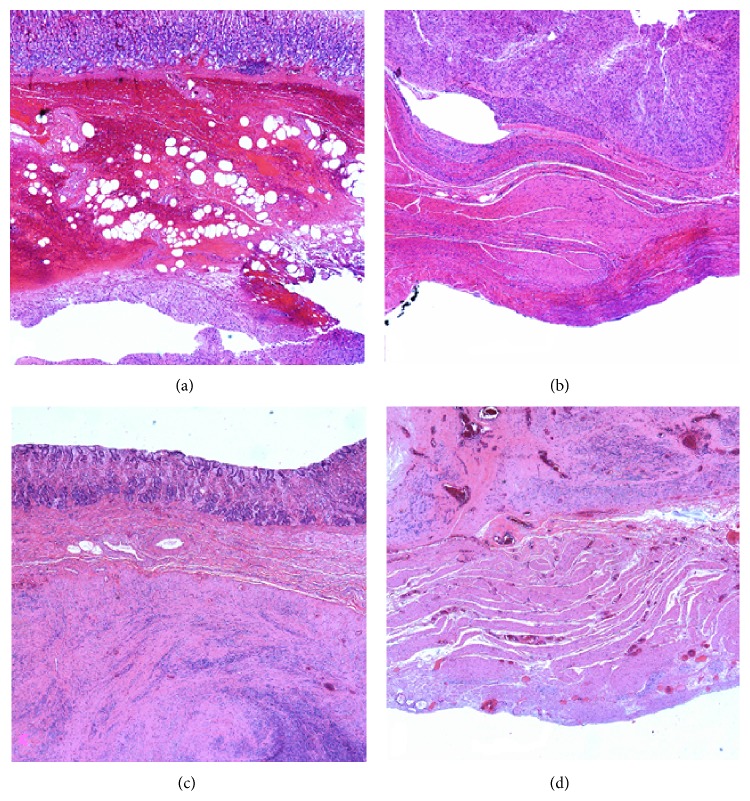
Patient 3's endoscopic specimen (a) shows spindle cells representative of GIST involving the submucosa and margin of the sample. The laparoscopic specimen (b) from the same patient demonstrates GIST cells confined superficial to the serosal surface. Patient 4's endoscopic specimen (c) likewise shows spindle cell involvement at the specimen's margin, while the laparoscopic specimen (d) exhibits a negative oncologic margin.

The third patient was found to have a suspicious lesion on imaging performed for dyspepsia, and subsequent EUS revealed a submucosal mass measuring 1.7 × 1.1 cm. Hybrid resection was considered due to the endophytic nature of the tumor and the poor visualization was expected with laparoscopy alone. Hybrid resection was successful, and pathology demonstrated a 2.6 × 1.9 cm mass with mitotic rate of 2 mitoses/50 hpf arising from the serosal layer (Figure 3(a)). The partial full thickness gastrectomy specimen demonstrated residual GIST in the resection site (Figure 3(b)). The final specimen had negative margins.

The final patient, after presenting with left upper quadrant pain, was found on EUS to have a heterogeneous, lobulated 4.5 × 3.0 × 2.6 cm endophytic submucosal mass arising in the cardia of the stomach, approximately 25 mm from the GE junction. The patient was treated with neoadjuvant imatinib for 2 months prior to surgery to reduce the size. The patient was considered for hybrid resection due to the location at the GE junction. Endoscopic resection was first performed with laparoscopic assistance (push). Full thickness resection site resection was then performed without constricting the GE junction. Pathology demonstrated a smaller mass than before, at 3.5 × 3.3 × 3.2 cm, which extended to the submucosal layer and had 0 mitoses/50 hpf (Figure 3(c)). The wedge resection specimen revealed residual GIST with negative margins (Figure 3(d)). There were no complications after any of the procedures and all patients were discharged from the hospital within 48 hours.

## 4. Discussion

GISTs are the most common mesenchymal tumors involving the gastrointestinal tract, with 50% occurring in the stomach [15]. While some GISTs are amenable to a straightforward laparoscopic wedge resection with little morbidity, endophytic lesions, tumors that originate in the cardia or fundus near the GE junction or near the pylorus, are significantly more challenging from an operative standpoint. Conversely, while many submucosal mass lesions may be removed endoscopically, GIST and other fourth-layer tumors are more difficult to resect endoscopically and, unlike more superficial lesions, may leave tumor cells behind with a positive deep resection margin. Despite one recent retrospective study showing no significant difference in recurrence between R1 and R0 resections [16], the current standard of care for proper surgical GIST management is a full thickness resection with negative margins. The hybrid push-pull laparoscopic and endoscopic approach presented here appears to be safe and effective in the resection of gastric GISTs in a small series across two institutions. The approach was applicable to lesions that arose from the muscularis propria layer and were positioned in locations that were problematic for straightforward surgical resection. This study demonstrates an oncologically sound, minimally invasive, and organ sparing approach to the management of select gastrointestinal stromal tumors.

GISTs remain a therapeutic challenge, as many require resection due to malignant potential, can be resistant to systemic therapy alone, and can occur in locations that are difficult for traditional surgical procedures [15, 17, 18]. Several techniques have been explored in recent years to address GISTs that cannot be removed by laparoscopic wedge resection. One method involves an intragastric approach, in which ports were introduced into the stomach through the abdominal wall with simultaneous endoscopic visual guidance [19]. This technique requires one or more gastrotomies. Another series describes a method in which the tumor is removed laparoscopically after traction sutures are placed according to endoscopic guidance to lift the desired area of the stomach [20]; however, this method could require resection of a large amount of surrounding gastric wall and may not be feasible for tumors near the GE junction or pyloric channel tumors. The hybrid method described here allows a safe, oncologically sound resection of endophytic tumors and tumors in difficult locations while minimizing the proportion of surrounding stomach which is resected. The push-pull technique also allows for precise localization of the tumor. A recent case series describes a technique in which endoscopists and laparoscopists cooperated to remove GIST tumors [21], where tumors <3 cm were removed endoscopically and tumors >3 cm were removed laparoscopically. The results of that study demonstrated that endoscopic resection alone, even for tumors less than 2 cm, left behind residual GIST at the deep margin. Another approach involved stenting the GE junction with an endoscope while a GIST was removed transgastrically [22], again requiring gastrotomy and subsequent closure following the laparoscopic resection of the tumor.

The present series supports previous work [7] that the hybrid approach can be applied for endophytic lesions in anatomically difficult locations for a standard laparoscopic wedge resection. Additionally, for all four tumors, GIST was found in the full thickness laparoscopic resection after endoscopic resection was complete, indicating that techniques such as endoscopic mucosal resection (EMR) and ESD may leave a positive margin with fourth-layer tumors. Due to the likelihood of a positive deep margin, EMR and ESD without a full thickness resection are better suited for lesions superficial to the muscularis propria layer [7].

Endoscopic resection of GIST tissue requires rupturing the capsule of the tumor, which surgical removal alone may be able to avoid. Endoscopic submucosal-mucosal resection has been shown, however, to remove tissue without recurrence at 21 months in a small sample of patients [23]. Further, EUS-guided biopsy is frequently used to diagnose gastric tumors, and this method has the same theoretical risks of spreading tumor as endoscopic resection if there is no perforation of the stomach. Laparoscopic resection for localized, resectable GISTs unfortunately has been estimated to have a recurrence rate of 50% at five years [24], which implies that there are other major factors governing recurrence other than rupture of the tumor capsule. Perforation of the stomach is a known risk with endoscopic tumor resection, and peritoneal seeding becomes a major concern if this occurs. The push-pull method may actually reduce this risk by allowing the endoscopist to focus just on extensive removal of the endophytic portion of the tumor rather than also removing the entire tumor base.

This study has several limitations. The study was retrospective and there was no concurrent control group in which to compare outcomes. The approach is applicable to a highly selected subset of tumors and is not suggested as a replacement for laparoscopic wedge resection for tumors in amenable locations. While the study was multi-institutional, the sample size was small. Additionally, the approach requires significant collaboration and coordination between two different specialties.

## 5. Conclusion

The hybrid push-pull approach presented here was safe and effective in the management of endophytic gastric GIST tumors in anatomically complex locations. Four patients underwent a minimally invasive, organ sparing resection with no postoperative morbidity. Pathologic evaluation of the full thickness laparoscopic resection specimens in this series demonstrated that tumor cells are left behind in the base following endoscopic resection of fourth-layer lesions. This data suggests that endoscopic approaches without a full thickness component may not be oncologically sound for gastric GIST tumors. In a highly selected subset of patients with endophytic GISTs in complex locations, the hybrid push-pull resection may represent a novel and improved approach to the current standard surgical management.

## Figures and Tables

**Table 1:** Baseline patient characteristics.

Pt^a^	Age/gender	Presenting symptoms	Size of resected mass (cm^b^)	Depth of invasion	Location of mass	Mitotic rate (per hpf^c^)	Reason for hybrid	Endoscopic specimen margin	Laparoscopic specimen margin	Duration (min^d^)
1	56/F^e^	Melena/anemia	3.0 and 2.8	Muscularis propria	Fundus (near GE^f^ junction)	1/50	Near GE junction and endophytic	Positive	Negative	209

2	57/M^g^	Dysphagia	4.2	Muscularis propria	Antrum, posterior wall	1/50	Endophytic; difficult to identify laparoscopically	Positive	Negative	157

3	75/M	Dyspepsia	2.6 × 1.9	Serosa	Body, anterior wall	2/50	Endophytic; difficult to identify laparoscopically	Positive	Negative	137

4	68/F	LUQ^h^ pain/chest pain	3.5 × 3.3 × 3.2	Submucosa	Cardia (near GE junction)	0/50	Near GE junction and endophytic	Positive	Negative	146

^a^Pt = patient.

^b^Cm = centimeter.

^c^Hpf = high-powered field.

^d^Min = minutes.

^e^F = female.

^f^GE = gastroesophageal.

^g^M = male.

^h^LUQ = left upper quadrant.
